# Abetalipoproteinemia: two case reports and literature review

**DOI:** 10.1186/1750-1172-3-19

**Published:** 2008-07-08

**Authors:** Rola Zamel, Razi Khan, Rebecca L Pollex, Robert A Hegele

**Affiliations:** 1 Department of Medicine and Biochemistry, University of Western Ontario, London, Ontario, Canada; 2 Robarts Research Institute, Schulich School of Medicine and Dentistry, University of Western Ontario, London, Ontario, Canada

## Abstract

Abetalipoproteinemia (ABL, OMIM 200100) is a rare, autosomal recessive disorder, characterized by fat malabsorption, acanthocytosis and hypocholesterolemia in infancy. Later in life, deficiency of fat-soluble vitamins is associated with development of atypical retinitis pigmentosa, coagulopathy, posterior column neuropathy and myopathy. ABL results from mutations in the gene encoding the large subunit of microsomal triglyceride transfer protein (MTP; OMIM 157147). To date at least 33 *MTP *mutations have been identified in 43 ABL patients. We describe the clinical progress of two patients, both currently in the fifth decade of life, who were diagnosed with ABL as children and were treated with high oral doses of fat soluble vitamins, including vitamin E over the last three decades. Treatment appears to have been associated with arrest of the neuropathy and other complications in both patients. Because pharmacologic inhibition of MTP is being developed as a novel approach to reduce plasma cholesterol for prevention of cardiovascular disease, defining the long-term clinical features of patients with a natural deficiency in MTP might provide some insight into the possible effects of such treatments. We review the range of clinical, biochemical and molecular perturbations in ABL.

## Background

Abetalipoproteinemia (ABL; OMIM 200100) is a rare metabolic disorder with a frequency <1 in 100,000. The clinical association of peripheral blood acanthocytosis with atypical retinitis pigmentosa and ataxia was first reported by Bassen and Kornzweig in 1950 [1]. In 1958, Jampel and Falls observed low serum cholesterol values in affected individuals [2] and in 1960 Salt noticed the absence of serum beta-lipoprotein in a patient with the syndrome. Consequently the name of the disease was changed to ABL [2]. Eventually, the fundamental biochemical defect was determined to be a complete absence of plasma apolipoprotein (apo) B-containing lipoproteins, namely chylomicrons, very-low density lipoprotein (VLDL) and low-density lipoprotein (LDL). In 1986, the *APOB *gene, its mRNA and the apo B content of the hepatocytes were found to be normal in ABL patients, suggesting that defective post-translational processing and secretion of apo B was the cause of ABL [3]. In 1992, a deficiency of microsomal triglyceride transfer protein (MTP) activity was suggested to be the proximal cause of ABL [4]. In 1993, the region on chromosome 4q22-24 that encodes the large subunit of MTP was cloned and sequenced, and human *MTP *mutations in ABL patients were reported [5].

The 894 amino-acid protein product of *MTP *(also called 97-KDa subunit) forms a heterodimer with the ubiquitous endoplasmic reticulum enzyme protein disulfide isomerase (PDI) [6]. MTP acts as a chaperone that facilitates the transfer of lipids onto apo B. The MTP molecule is predicted to consist of three major structural domains: N-terminal β-barrel (residues 22–297), middle α-helical (residues 298–603) and C-terminal domains (residues 604–894). The N-terminal β-barrel domain mediates the interaction with the N terminus of apo B; the middle α-helical domain mediates the interaction with both PDI and apo B; and the C-terminal mediates the lipid-binding and transfer catalytic activity of MTP.

Mutations that lead to the absence of a functional 97-KDa subunit cause ABL. We describe the medical history of two patients with ABL and summarize the range of reported mutations and their associated clinical phenotypes.

## Case presentations

### Case #1

A 46 year old female of Northern European descent (patient 1 in reference [7] and patient 27 in Additional file 1) was diagnosed with ABL at age 11, when she presented with right ptosis. Medical work-up revealed acanthocytosis on peripheral blood smear, which at the time was pathognomonic for a diagnosis of ABL. Her ptosis was corrected surgically. In retrospect, she had a history of diarrhea in infancy that resolved upon fat restriction. She was noted to have ataxia and paresthesia in a glove-and-stocking distribution in her teenage years. She was started on high oral doses of fat-soluble vitamins approximately 9 years after her diagnosis. In 1999, the molecular basis of her condition was found to be a homozygous frameshift mutation in exon 13 of *MTP *due to a single nucleotide base-pair deletion (c.1820delG) [7].

At age 26 she married. At age 34, she gave birth to a full-term healthy male infant. During her pregnancy, she was advised to stop all intake of vitamin A to avoid the potential risk of vitamin A teratogenicity, despite the fact that her serum beta-carotene concentration was below the lower limit of the normal range. Postpartum, she developed a right corneal ulcer that required corneal transplantation after a year. She was restarted on vitamin A postpartum. Her corneal transplant failed and currently she is waiting for a repeat right corneal transplant. Of note, she was not able to achieve a second pregnancy despite multiple attempts. Her past medical history included scalp basal cell carcinoma successfully treated with cryotherapy. She also had a long history of oligomenorrhea. Subjectively, her symptoms had otherwise remained stable for decades. Her parents were first cousins. Her family history included two sisters with Alport syndrome. There was no family history of ABL.

She was followed yearly and evaluated in 2007, 34 years after her initial diagnosis. Her medications included vitamin K 10 mg twice a week, calcitriol 0.25 mcg daily, beta-carotene 40,000 IU daily, vitamin A 10,000 IU daily, vitamin E 400 IU daily, vitamin B6, vitamin B12, calcium, magnesium and eye drops.

On physical exam her weight was 56.5 kg. She had mild bilateral scleral icterus. Cranial nerve exam was normal apart from decreased visual acuity on the right side. The gait was wide-based. Romberg test was positive but she had no dysmetria and no dysdiadochokinesia. Sensory exam was remarkable for decreased vibration sense in upper and lower limbs and decreased position sense in the lower limbs. Pain sensation was normal. Motor exam showed normal tone and power in major muscle groups with absent knee reflexes. The Babinski sign was negative. Cardiovascular exam was unremarkable, specifically the heart rhythm was regular and there was no evidence of congestive heart failure. The rest of the physical examination was unremarkable.

**Figure 1:**
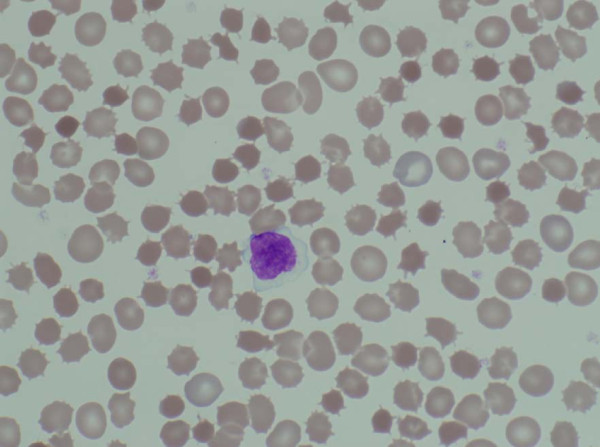
Acanthocytes on the peripheral blood smear of case #1.

Lab evaluation included a normal white blood cell count, normal hemoglobin concentration, normal mean red blood cell volume with a slightly elevated reticulocyte count at 112 × 10^9^/L (normal 10 to 100 × 10^9^/L). The blood film showed acanthocytosis (Figure 1). Serum concentrations of 1,25-dihydroxy-vitamin D and ionized calcium were normal. Serum vitamin E was low at <5 μmol/L (normal 18–29 μmol/L). International normalized ratio of prothrombin time was normal at 1.1. Total bilirubin was slightly elevated at 27.7 μmol/L (normal 3.4–17.1 μmol/L), while direct bilirubin, serum transaminases, gammaglutaryl transferase, albumin and total protein were normal. Serum creatine kinase was elevated at 211 mmol/L (normal 26–140 mmol/L). Serum thyrotrophin, progesterone and 17-OH-progesterone were within normal limits for age and sex. No changes in treatment were recommended.

### Case #2

In 1976, a 16-year old girl presented with progressively worsening coordination. During infancy, her persistent diarrhea resolved with the institution of a low-fat diet. At age 11, routine blood tests revealed acanthocytosis with undetectable plasma cholesterol and triglyceride, which together with normal parental lipid profiles suggested a diagnosis of ABL. Oral vitamin A 25 000 IU daily, 25-hydroxy vitamin D 10 000 IU daily, and vitamin K 5 mg daily were prescribed. At age 16, she began to experience generalized weakness and impaired balance. Neurological examination revealed mild dysarthria, reduced muscle bulk, bilateral proximal muscle weakness, absent deep-tendon reflexes, upgoing plantar reflexes, reduced sensitivity to light touch with loss of proprioception and vibration sense in a glove-and-stocking distribution, mild intention tremor, dysdiadochokinesia in upper and lower limbs, a wide-based ataxic gait and positive Romberg sign [8]. Evoked motor unit potentials in the right extensor digitorum brevis were decreased (30% of the lower limit of normal) and electron microscopy of the sural nerve showed a marked reduction in large myelinated fibres [8]. Plasma creatine kinase was 314 IU/L (normal <45 IU/L). Plasma triglycerides and cholesterol were markedly decreased (1.8 [normal 5.2] and <0.1 [normal 1.3] mmol/L, respectively) while VLDL and LDL fractions were absent on lipoprotein electrophoresis. Plasma, erythrocyte and adipose tissue vitamin E levels were undetectable. Vitamin E deficiency was diagnosed. High-dose oral vitamin E therapy at 800 mg/day was added to her treatment in 1981, and was progressively titrated to her current dose of 14 400 IU (220 IU/kg).

She was followed yearly and re-evaluated 26 years later in 2007 at age 47. Her health had remained good and she remained compliant with treatment. Sequencing of the *MTP *gene indicated that she was a compound heterozygote for known pathogenic mutations c.2237G>A (p.G746E) and c.2524A>T (p.K842X) [7]. Neurological findings had either improved or not progressed. Examination revealed no dysarthria with normal muscle bulk and strength. Both tremor and dysdiadochokinesia had dissipated. Gait had improved to the point that she could run. However, deep-tendon reflexes remained absent and plantar reflexes were still upgoing. Light touch, vibration and proprioreception deficient and bilateral dysmetria persisted, but had not progressed. Evoked motor potentials in the right extensor digitorum brevis had improved to 75% of the lower limit of normal. Electromyography of sural and ulnar nerves indicated a mild sensory neuropathy. Serum CK was normal, as were beta-carotene and prothrombin time, while vitamin E was 6 μmol/L (normal 11–46 μmol/L).

## Discussion

ABL is a rare autosomal recessive metabolic disorder with multi-system manifestations. All reported patients have fat malabsorption, acanthocytosis, low serum cholesterol and deficiency of serum apo B. Retinitis pigmentosa, spinocerebellar ataxia and myopathy have complicated most of the cases. Two reports of possible association with certain cancers like ileal adenocarcinoma and metastatic spinal cord glioblastoma [9,10] have been reported and fatty liver was reported in 4 cases. The clinical presentation is very heterogeneous and previous reports had suggested that MTP deficiency is not the sole cause of ABL [11].

**Figure 2:**
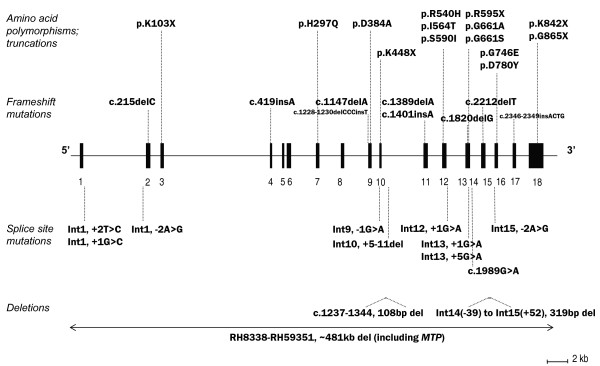
**Genetic map of MTP mutations in patients with abetalipoproteinemia**. The map shows the genomic structure of MTP gene. Black boxes represent exons; dotted lines point to mutation positions. Above the map are single-nucleotide and small insertion-deletion mutations. Nucleotide position in reference to the first ATG of MTP cDNA (GenBank Accession No. NM_000253) are shown. Directly under the linear map are splicing mutations. The position relative to the intron-exon boundary is shown numerically. Larger deletions are shown as horizontal lines below the linear map, with the span of the deleted region indicated by text label.

ABL is characterized by absent plasma apo B-containing lipoproteins that results from *MTP *mutations [12]. To date at least 33 *MTP *mutations have been identified in 43 ABL patients (Figure 2). Absorption of fats and fat-soluble vitamins are compromised, leading to failure to thrive and fat-soluble vitamin deficiency [13]. Vitamin E deficiency is associated with hyporeflexia, reduced proprioception and vibratory sensation, muscle weakness and a Friedrich's-like ataxia [14]. Before the use of high-dose oral fat-soluble vitamin treatment, many ABL patients developed neurological complications before the second decade and some did not survive past the third decade [13].

Homozygous dysfunctional mutations in *APOB *lead to a clinically similar disorder called homozygous hypobetalipoproteinemia (HHBL, OMIM 107730). While obligate heterozygote parents of HHBL patients have half-normal plasma levels of apo B and LDL-cholesterol, obligate heterozygote parents of ABL patients have normal plasma lipoprotein profiles. HHBL patients receive similar treatment advice as ABL patients.

Muller and co-workers [15] first reported in 1974 that high dose oral vitamin E (100 IU/kg) could increase undetectable serum vitamin levels in ABL patients. Thereafter, short-term efficacy of high-dose oral vitamin supplements was noted [8,16]. Reports of longer-term treatment in ABL have been limited. For instance, combined vitamin E and A therapy initiated before age 2 was reported to markedly attenuate retinal degeneration 10 years later [17]. Twelve-year follow-up indicated that high dose oral vitamin E (100 IU/kg) slowed retinal degeneration [18]. Long-term high dose vitamin E reduced neurological sequelae in other vitamin E-deficient diseases, such as chronic cholestasis [19], autosomal-recessive vitamin E deficiency (AVED) [20] and short-bowel syndrome [21]. Our observations over more than a quarter-century suggest that ultra-long-term high-dose oral vitamin therapy including vitamin E is associated with arrest of the neurological complications in at least some patients with ABL. Such long-term experience in ABL is relevant particularly since pharmacological MTP inhibition has recently been proposed as a therapeutic option for patients with severe hypercholesterolemia [22]. Interestingly, coronary arteries of ABL patients appear to be free of atherosclerotic lesions [23].

Gastrointestinal manifestations of ABL include diarrhea and fat soluble vitamin deficiency, which are consistent features in all reported cases. These manifestations usually develop during infancy and are worsened with a diet rich in fat. The diarrhea subsides later in part because patients learn to avoid fatty foods. However, the low serum levels of fat soluble vitamins continue, because the plasma transport and delivery of these vitamins to tissues depends almost exclusively (for vitamin E and beta-carotene) or in part (for vitamins A, D, and K) on intact synthesis and secretion of apo B-containing lipoproteins. Supplementation with high dose vitamin E results in increased serum vitamin levels to not more than 30% of the lower limit of normal. On the other hand, high doses of vitamin A therapy can normalize serum levels. This reflects the fact that despite impaired absorption and transport from the intestine, subsequent transport of vitamin A in plasma by retinol-binding protein is not impaired in ABL.

Hepatic manifestations of ABL include elevated serum transaminases with hepatomegaly due to hepatic steatosis [24,25], although neither of the two patients reported here had these findings. Cirrhosis has been reported in a few cases and one patient required transplantation for hepatic cirrhosis; post transplantation the serum lipoprotein profile increased to normal levels, however the fat absorption defect continued as the mutant MTP remains expressed in the intestine [26]. Liver biopsies in patients with ABL have shown marked steatosis that may be reflected in raised serum transaminase concentrations [27].

Hematologic manifestations of ABL include acanthocytosis. These abnormal shaped cells comprise 50% or more of circulating erythrocytes and were among the earliest laboratory features of the disorder (see Figure 1). Their structure inhibits rouleaux formation, leading to extremely low erythrocyte sedimentation rates. Anemia has been reported in some cases of ABL [28]. The likely cause was deficiencies of iron, folate, and other nutrients secondary to fat malabsorption. Hemolysis which appears to result from accelerated hydroperoxidation of fatty acids secondary to tocopherol deficiency may also contribute to anemia [24]. Elevated prothrombin time, and international normalized ratio due to vitamin K deficiency, was reported in several cases [24]. In two cases, significant gastrointestinal bleeding associated with severe vitamin K deficiency was present in infancy or childhood [24].

Neurological involvement in ABL may be the most serious clinical manifestation. In ABL both central and peripheral nervous systems are affected; patients can have either upper or lower motor neuron findings or both. The primary driving pathology is demyelination [25]. The onset of neurologic disease usually begins in the first or second decade of life and in the past often progressed to catastrophic disability, although some patients inexplicably escaped serious affliction until much later in life [25]. The long-term clinical results of vitamin E therapy from multiple previous studies show improvement in neurologic dysfunction with vitamin E treatment and early therapy before the age of 16 months prevents neurologic dysfunction [8,16]. In both cases we have presented, high-dose oral vitamin E replacement appeared to be associated with long-term stabilization of neurologic status, although we appreciate the anecdotal – and perhaps non-generalizable – nature of this observation.

Muscle involvement in ABL affecting both striated and smooth muscle has been reported in some patients, and furthermore was the cause of premature death cases among a few ABL patients [28-30]. While the etiology of myopathy is unclear, myositis appeared to be related to ceroid pigment deposition, while muscle weakness could possibly be related to vitamin E deficiency and neuropathy. A quadriceps muscle biopsy performed on a 26 year old male ABL patient who presented with sudden onset of severe weakness revealed ceroid pigment in the muscle fibers [30]. The etiology of myopathy in that case remains unclear due to preserved myofilaments, a feature not consistent with myopathy due to vitamin E deficiency [30]. Death related to cardiomyopathy has been reported in a 10 year old male ABL patient and a 36 year old female ABL patient [28,29]. Autopsy of the male patient showed perinculear deposits of lipochrome pigment in cardiomyocytes, suggesting tocopherol deficiency. A male patient of Northern European descent (Patient 8 in Additional file 1), died at the age of 18 due to respiratory failure related to neuropathy/myopathy; no autopsy findings were reported.

Ophthalmic involvement in ABL is variable and appears to cover a wide range of symptoms and ophthalmic manifestations [31]. The most prominent abnormality is pigmentary retinal degeneration. Most patients have loss of night vision early in the course of disease, while some patients also present with loss of color vision. The retinopathy often produces slowly enlarging annular scotomas with macular sparing, such that patients are relatively unaware of the progression of the disease. Complete loss of vision can ultimately occur [31]. Fundoscopic examination reveals an atypical pigmentation of the retina characterized by small, irregularly distributed, white spots. Electroretinogram and fluorescein angiography investigations have shown the retina to be affected in asymptomatic ABL patients [31].

The mechanism underlying the retinopathy in ABL is not clear. Results from previous studies are inconsistent. A report of results of up to 18 years of follow-up of six ABL patients showed that high dose oral vitamin E therapy (47–172 mg/kg/day) not only prevented the development of retinopathy but also appeared to arrest the progression of retinopathy [18]. In 2001, another study of 10 ABL patients followed for a mean of 11.7 years showed that combined vitamin A and E supplementation that was initiated prior to 2 years of age markedly attenuated the severe retinal degeneration. Yet, fundoscopic and functional retinal changes can occur despite early treatment. Of note, all ABL patients who received vitamin therapy prior to 2 years of age were free of the neurologic and systemic complications that are usually associated with untreated ABL [17]. Other reported abnormalities include, ophthalmoplegia, anisocoria, nystagmus, strabismus and ptosis. Although the pathogenetic mechanism for these symptoms is unclear, a neurological basis was suggested.

In addition to ptosis, case #1 developed a unilateral corneal ulcer after oral vitamin A was discontinued. However, her symptoms did not improve following re-institution of vitamin A supplementation. A previous report described an ABL patient with mild xerophthalmia [32]. Corneal ulceration is unusual among ABL patients.

It is of interest that reproductive system manifestations seem to be rather minimal in ABL, indicating that in the absence of LDL, there is sufficient capacity for synthesis and secretion of steroid hormones, including sex steroids, provided by HDL. One study evaluating the endocrine function in a 37 year old female ABL patient of Greek origin who was diagnosed at age 5 years after instituting dietary modifications, found that serum progesterone and 17 (OH) progesterone are low at both exams as well as serum progesterone at day 21 of the menstrual cycle was below normal [33]. In addition, in context of low serum cholesterol one might expect a low fertility rate among ABL patients. However, successful pregnancy has been reported on several occasions, as seen with case #1 [31,34,35].

### Prognostic factors predicting disease course

Previous case reports have variably suggested links between age at diagnosis, onset of treatment with low fat diet and vitamin replacement therapy, type of *MTP *mutation and *APOE *genotype with the long-term outcome of patients with ABL.

Regarding prognosis based upon age at diagnosis, many ABL patients present in the 2^nd ^to 4^th ^decades, while a few others present in the 1^st ^and 6^th ^decades. It is possible that an earlier presentation is due to a more severe phenotype and perhaps a worse outcome, which would be more refractory to treatment. However, a later presentation might also be associated with a more severe outcome due to a longer period of untreated vitamin deficiency, especially during growth and development.

Regarding the potential relationship between the type of *MTP *mutation and long-term outcome, the current paucity of information makes it difficult to predict outcomes based on *MTP *genotype (Additional file 1). Also, regarding a possible relationship between *APOE *genotype and clinical progression of disease, a recent meta-analysis identified a linear relationship of *APOE *genotype with plasma LDL cholesterol [36]. On the background of *APOE *E2 homozygosity, some missense mutations of *MTP *with mild to moderate effects on protein structure and function have been suggested to lower plasma LDL cholesterol levels more dramatically (see patient 19 in Additional file 1) [37]. In another report that included information about *APOE *genotype in six ABL patients, the E4/E2 genotype seemed to be associated with less favourable outcomes [7].

### Long term treatment and follow-up of ABL

The current standard treatment for ABL is dietary modification and replacement of fat soluble vitamins. A low fat diet has been shown to improve steatorrhea associated with fat malabsorption and allow absorption of other nutrients essential for growth and development. High dose oral fat soluble vitamin supplementation has anecdotally been associated with improved clinical outcomes. High dose oral vitamins are thought to bypass the intestinal chylomicron assembly pathway via the portal circulation (medium-chain triglyceride pathway).

Oral vitamin E is typically given in daily doses ranging from 2400 to 12000 IU. Plasma vitamin E levels might not accurately reflect the whole body content of vitamin E and thus the adequacy of vitamin replacement may be difficult to gauge from serum concentrations [38]. In one study, serum vitamin E concentrations, which were initially undetectable in all ABL patients, became measurable after oral supplementation, although they never reached the normal range [16]. Furthermore, needle aspiration of adipose tissue showed that high doses of vitamin E (150 mg/kg body weight daily) were associated with increased tissue levels of α-tocopherol in almost all ABL patients. This was associated with ameliorated neurologic symptoms in older subjects and possible protection from neurologic pathology, if supplement is started early enough [38]. Therefore, the serum vitamin E level can be used to monitor compliance and adequacy of therapy.

High doses of vitamin A (100–400 IU/kg/day) are needed to alleviate the deficiencies; the dosing can be variable and titration can be guided by serum carotene concentrations. Vitamin A toxicity was reported in one patient. This patient developed papilledema a few days after introduction of vitamin A therapy, while her serum vitamin A level was within normal limits [39].

Vitamin D deficiency is not consistently described among ABL patients. However, low serum ionized calcium, vitamin D and bony abnormalities have been described [33], and thus vitamin D replacement (1000 mg daily) should be considered in all ABL patients. An abnormal coagulation profile with prolonged prothrombin time and increased international normalized ratio has been reported in many ABL patients: two patients had severe gastrointestinal bleeding. In addition vitamin E absorption can exacerbate the deficit in vitamin K therefore, replacement of vitamin K is necessary (5 mg daily). Other supplementary nutrients like iron, folic acid can also be considered. Patients need to be followed regularly for evaluation of symptoms and complications, and to monitor compliance with therapy.

## Conclusion

In summary, ABL is a rare disease of lipoprotein metabolism that has drawn attention to the importance of MTP in the assembly and secretion of apo B-containing lipoproteins. Without treatment, ABL symptoms can be debilitating in most of the patients and life expectancy is expected to be reduced. Current evidence suggests that early treatment with high oral doses of combined vitamin A and E, if introduced early enough, can reduce the potential severity of neuropathy and retinopathy. Furthermore, anecdotal evidence, such as the history of the two patients reported here, suggests that vitamin replacement treatment can reduce morbidity and delay early mortality associated with ABL. There is still a need for novel therapeutic approaches to ABL, since vitamin therapy alone is not sufficient to completely control or cure this disease.

## Consent

Written informed consent was obtained from the patients for publication of these case reports and any accompanying images. Copies of the written consent are available for review by the Editor-in-Chief of this journal.

## Competing interests

The authors declare that they have no competing interests.

## Authors' contributions

RZ, RK and RAH conceived of the study, interpretation of results and manuscript writing. RLP assisted with manuscript writing, editing the manuscript and created the genetic map. All authors read and approved the final manuscript.

## Supplementary Material

Additional file 1 Reported *MTP *mutations and their clinical phenotypes. The data provided represent the reported *MTP *mutations in patients with ABL and the clinical findings of these patients. Click here for file
