# Supplementary material for: Abetalipoproteinemia: two case reports and literature review
Source: Orphanet J Rare Dis. 2008 Jul 8;3:19. doi: 10.1186/1750-1172-3-19 (PMC2467409; doi:10.1186/1750-1172-3-19)
Supplement: Additional file 1 — Reported MTP mutations and their clinical phenotypes. The data provided represent the reported MTP mutations in patients with ABL and the clinical findings of these patients. [file 1750-1172-3-19-S1.doc]

**Additional file 1. Reported *MTP* mutations and their clinical phenotypes**

| **ID** | **Ref** | **Age at Dx (yr)** | **Age**  **(yr)** | **Ethnicity** | **Sex** | **Mutation(s)** | **Consequence** | **Presenting**  **symptoms** | **Gastro-intestinal**  **involvement** | **Acanthocytosis** | **Ophthalmic involvement** | **Neurological**  **findings** | **Other** |
| --- | --- | --- | --- | --- | --- | --- | --- | --- | --- | --- | --- | --- | --- |
| 1 | [16] | N/A | 9 | North  African,  Jewish | F | Homozygous,  c.307A>T | Nonsense mutation (K103X) | N/A | + | + | +  Abn ERG & EOG | N/A | N/A |
| 2 | [12] | N/A | 14 | N/A | F | Homozygous,  c.1783C>T | Nonsense mutation (R595X) | N/A | N/A | N/A | N/A | N/A | N/A |
| 3 | [17] | N/A | 57 | Ashkenazi  Jewish | M | Homozygous,  c.2593G>T | Nonsense mutation (G865X) | N/A | + | + | +  Abn ERG & EOG, pigmentary changes | N/A | N/A |
| 4 | [40] | N/A | 31 | Ashkenazi  Jewish | F | Homozygous,  c.2593G>T | Nonsense mutation (G865X) | N/A | + | + | +  Abn ERG & EOG | N/A | N/A |
| 5 | [40] | N/A | 29 | Ashkenazi  Jewish (brother of 3) | M | Homozygous,  c.2593G>T | Nonsense mutation (G865X) | N/A | + | + | +  Abn ERG & EOG | N/A | N/A |
| 6 | [40] | N/A | 27 | Ashkenazi  Jewish | M | Homozygous,  c.2593G>T | Nonsense mutation (G865X) | N/A | + | + | +  Abn ERG & EOG | N/A | N/A |
| 7 | [40] | N/A | 17 | Ashkenazi  Jewish (brother of 5) | M | Homozygous,  c.2593G>T | Nonsense mutation (G865X) | N/A | + | + | +  Abn ERG & EOG | N/A | N/A |
| 8 | [7] | 12 | 18 (died) | Northern  European | M | Homozygous,  c.2593G>T | Nonsense mutation (G865X) | N/A | + | + | +  Severe retinopathy | +  Severe ataxia, areflexia | *APOE* E4/2 |
| 9 | [6] | 0.4 | N/A | Chinese | M | Homozygous, Int10 +5-11 delGTGCAAA | Splice site mutation | N/A | + | N/A | N/A | - | N/A |
| 10 | [5] | N/A | N/A | N/A | F | Homozygous,  Int13 +5G>A | Splice site mutation | FTT | N/A | N/A | N/A | N/A | N/A |
| 11 | [6] | Dx from cord blood | N/A | British | N/A | Compound heterozygote, Int13 +1G>A; c.1989G>A | Splice site mutations | N/A | N/A | N/A | N/A | N/A | N/A |
| 12 | [41] | 29 | N/A | Japanese | M | Homozygous, Int9 -1G>A; UPD (mother) | Splice site mutation | Identified during routine exam | +  Diarrhea, anemia, short stature | + | +  Childhood loss of night vision, retinopathy | + | N/A |
| 13 |  | N/A | 22 | Iranian Jewish | M | Homozygous, Int1 -2A>G | Splice site mutation | N/A | + | + | +  Abn ERG & EOG | N/A | N/A |
| 14 | [40] | N/A | 14 | Iranian Jewish (cousin of 13) | M | Homozygous, Int1 -2A>G | Splice site mutation | N/A | + | + | +  Abn ERG & EOG | N/A | N/A |
| 15 | [7] | 17 | 31 | Northern  European | M | Homozygous,  c.1619G>A | Missense mutation (R540H), interferes with association between 97kDa and PDI | N/A | + | + | +  Severe retinopathy | +  Severe ataxia, areflexia | N/A |
| 16 | [7] | 24 | 44 | Northern  European | M | Homozygous, c.1769G>T | Missense mutation (S590I) | N/A | + | + | +  Severe retinopathy | - | N/A |
| 17 | [11] | 27 | N/A | Japanese | M | Homozygous,  c.2338A>T | Missense mutation (D780Y),  binds PDI but displays no activity | Identified during routine exam | -  Mild fatty liver | + | - | - | N/A |
| 18 | [9] | 52 | N/A | African/  Anglo-Saxon | M | Homozygous,  c.1769T>G | Missense mutation (S590I) | Identified during routine exam | + | + | + | -  Normal vitamin E without Rx | T2DM ; ileal adenoca |
| 19 | [37] | 19 | N/A | Italian | M | Compound heterozygote, c.1151A>C & c.1982G>C | Missense mutations (D384A and G661A), likely loss of transfer protein function | Obesity, HSM, abn OGTT, minimal LDL lowering | -  Mild steatorrhea | N/A | N/A | - | Serum vitamin A,K,E normal without Rx |
| 20 | [12] | N/A | 39 | N/A | F | Homozygous,  c.215delC | Frameshift mutation, early truncation | N/A | N/A | N/A | N/A | N/A | N/A |
| 21 | [6] | 1.6 | N/A | British | N/A | Homozygous,  c.419insA | Frameshift mutation, early truncation | Steatorrhea | + | N/A | N/A | +  Absent DTR,  ↓vibration | N/A |
| 22 | [6] | 25 | N/A | Dutch | N/A | Homozygous,  c.1147delA | Frameshift mutation, early truncation | Neuropathy | +  Intolerance to fatty food | N/A | +  Retinopathy at 25yr | +  Degeneration at 14yr, ataxia at 25yr | N/A |
| 23 | [6] | 0.9 | N/A | Irish | N/A | Compound heterzygote, c.419insA & c.1401insA | Frameshift mutations, early truncation | N/A | N/A | N/A | N/A | - | N/A |
| 24 | [6] | 0.6 | N/A | American | N/A | Compound heterozygote, c.419insA & Int13, +5G>A | Frameshift mutation; splice site mutation | N/A | N/A | N/A | N/A | - | N/A |
| 25 | [6] | 1.1 | N/A | Ashkenazi  Jewish | N/A | Homozygous, c.2212delT | Frameshift mutation, early truncation | Steatorrhea | + | N/A | N/A | +  Areflexia, hypotonia | N/A |
| 26 | [6] | 0.1 | N/A | Irish | N/A | Homozygous, Int13 +5G>A | Splice site mutation | Steatorrhea | N/A | N/A | N/A | - | N/A |
| 27 | [7] | 11 | 35 | Northern  European | F | Homozygous,  c.1820delG | Frameshift mutation, early truncation | Ptosis | + | + | - | - | *APOE* E4/3 |
| 28 | [11] | 32 | N/A | Japanese | F | Homozygous,  c.1389delA | Frameshift mutation, early truncation | Identified during routine exam | + | + | +  Fine mottling in the retina | +  Areflexia,  positive Romberg’s | N/A |
| 29 | [11, 35] | 15 | N/A | American | M | Homozygous,  c.419insA | Frameshift mutation, early truncation | Ataxia | + | N/A | +  Retinopathy at 22yr | +  Areflexia, ataxia, positive Romberg’s, dysmetria | Became a father of a healthy boy |
| 30 | [9] | N/A | N/A | French  Canadian | F | Homozygous, c.419insA | Frameshift mutation, early truncation | N/A | N/A | N/A | N/A | N/A | N/A |
| 31 | [37] | 3 | N/A | Italian | M | Homozygous,  c.1228-1230delCCCinsT | Frameshift mutation, early truncation | FTT, diarrhea at 2 months | +  ↑ Liver enzymes | + | N/A | - | *APOE* E2/3 |
| 32 | [40] | N/A | 24 | Ashkenazi  Jewish | M | Homozygous,  c.2212delT | Frameshift mutation, early truncation | N/A | + | + | +  Abn ERG & EOG | N/A | N/A |
| 33 |  | N/A | 22 | Ashkenazi  Jewish (brother of 32) | M | Homozygous,  c.2212delT | Frameshift mutation, early truncation | N/A | + | + | +  Abn ERG & EOG | N/A | N/A |
| 34 | [42] | 3.75 | N/A | Asian | F | Homozygous,  c.2346-2349insACTG | Frameshift mutation, early truncation | FTT | +  Diarrhea in infancy | + | N/A | +  Absent DTR | Skin & chest infection since infancy |
| 35 | [29] | 13 | 32 | Australian | F | Compound heterozygote,  Int1 +2T>C & c.419insA | Splice site mutation; frameshift mutation | Low serum apo B | +  ↑ AST, ALT, GGT, ALP | + | -  Mild xerophthalmia, no retinopathy | +  Reduced reflexes, disequlibrium at night | Dry skin |
| 36 | [7] | 8 | 24 | Northern  European | F | Compound heterozygote, c.1342A>T & Int12 +1G>A | Nonsense mutation (K448X); splice site mutation | N/A | + | + | - | - | *APOE* E3/3 |
| 37 | [11, 35] | 0.6 | N/A | American | M | Compound heterozygote, c.1783C>T  & Int15 -2A>G | Nonsense mutation (R595X); splice site mutation | FTT | +  ↑ Liver size at 2yr, MCT diet at Dx, DC at 2yr, N liver size at 4yr | + | +  Retinitis pigmentosa at 4yr | +  ↓ reflexes at 2yr, vitamin Rx at Dx | N/A |
| 38 | [7] | 16 | 40 | Northern  European | F | Compound heterozygote, c.2237G>A & c.2524A>T | Missense mutation (G746E), affects residues that are important for association with PDI; nonsense mutation (K842X) | N/A | + | + | +  Retinopathy | +  Severe ataxia and areflexia | *APOE* E3/3 |
| 39 | [37] | 39 | N/A | Italian | F | Compound heterozygote, Int9 -1G>A; failed to identify the 2^nd^ mutation | Splice site mutation | N/A | +  Diarrhea | N/A | - | - | ↓ iron, anemia, hepato-megaly, *APOB* & *SARA2* genes N,  *APOE* E4/4 |
| 40 | [43] | 1.25 | 5 | Japanese | M | Compound heterozygote,  Int1 +1G>C & c.1692T>C | Splice site mutation; missense mutation (I564T) | ↑ liver size & enzymes | - | - | - | -  vitamin E 0.43mg/dl (N 0.75-1.41) | N/A |
| 41 | [44] | N/A | N/A | N/A | N/A | Compound heterozygote, Allele 1: inframe del c.1237-1344; Allele 2: c.891C>G, c.1151A>C, c.1619G>A | Exon 10 deletion; Missense mutations (H297Q, D384A, R540H) | N/A | N/A | N/A | N/A | N/A | N/A |
| 42 | [37] | 0.25 | 10 | Muslim Arab | F | Homozygous,  ~481kb deletion between markers RH8338 and RH59351 | Contiguous deletion includes *MTP* and eight other genes | Diarrhea, FTT, anorexia | + | + | +  Abn ERG & EOG | N/A | Dysmor-physm, short stature, develop delay |
| 43 | [45] | 0.4 | N/A | Lebanese | M | Homozygous, inframe del Int14(-39) to Int15(+52) and also c.1981G>A | Exon 15 deletion;  missense mutation (G661S) | Diarrhea, FTT, ↑ liver enzymes | + | + | N/A | +  Hypotonia | ↑ liver size & enzymes, muscle wasting |

Abbreviations: (+) abnormality present; (-) abnormality absent; abn, abnormal; ERG, electroretinograms; FTT, failure to thrive; EOG, electrooculograms; Ref, reference; MCT, medium chain triglyceride; Dx, diagnosis; DC, discontinued; N, normal; N/A, not available; UPD, uniparenteral disomy; DTR, deep tendon reflexes; AST, aspartate aminotransferase; ALT, alkaline aminotransferase; GGT, gamma-glutamyltransferase; ALP, alkaline phosphatase; HSM, hepatosplenomegaly; T2DM, type 2 diabetes.

Note: The carrier frequency of c.2593G>T mutation among Ashkenazi Jews is 1:131, this carrier frequency suggests an ABL incidence rate of approximately one per 69,000 in this population [40].
